# Clearing the Air: Conflicts of Interest and the Tobacco Industry’s Impact on Indigenous Peoples

**DOI:** 10.1093/ntr/ntab267

**Published:** 2021-12-20

**Authors:** Raglan Maddox, Michelle Kennedy, Andrew Waa, Ali Drummond, Billie-Jo Hardy, Claradina Soto, El-Shadan Tautolo, Emily Colonna, Heather Gifford, Hershel Clark, Juliet P Lee, Patricia Nez Henderson, Penney Upton, Shane Kawenata Bradbrook, Shavaun Wells, Sydney A Martinez, Tom Calma

**Affiliations:** Modewa Clan, Papua New Guinea; National Centre for Epidemiology and Population Health, Australian Nation University, Canberra, Australian Capital Territory, Australia; Wiradjuri; School of Medicine and Public Health, University of Newcastle, Callaghan, NSW 2308, Australia; Hunter Medical Research Institute, Newcastle, NSW 2305, Australia; Ngāti Hine/Ngāpuhi; Eru Pomare Māori Health Research Unit, University of Otago, Wellington, Aotearoa, New Zealand Eru Pomare Māori Health Research Unit, University of Otago, Wellington, Aotearoa, New Zealand; Meriam and Wuthathi; Queensland University of Technology (QUT), Brisbane, Queensland, Australia; Dalla Lana School of Public Health, University of Toronto, Toronto, Ontario, Canada; Navajo/Jemez Pueblo; Department of Population and Public Health Sciences, Keck School of Medicine, University of Southern California, Los Angeles, CA, USA; Samoa/Ngāti Tapuniu; AUT Pacific Health Research Centre, Auckland University of Technology, Auckland, New Zealand; National Centre for Epidemiology and Population Health, Australian Nation University, Canberra, Australian Capital Territory, Australia; Ngāti Hauiti; Whakauae Research Services, Research for Māori Health & Development, Whanganui, Aotearoa, New Zealand; Navajo Nation (Diné); Black Hills Center for American Indian Health. Rapid City, SD, USA; Pacific Institute for Research and Evaluation-California, Prevention Research Center. Berkeley, California, USA; Navajo Nation (Diné); Black Hills Center for American Indian Health. Rapid City, SD, USA; Health Research Institute, University of Canberra, Canberra, ACT, Australia; Ngāi Tāmanuhiri, Rongowhakaata, Ngāti Kahungunu. Wellington, AotearoaNew Zealand; National Centre for Epidemiology and Population Health, Australian Nation University, Canberra, Australian Capital Territory, Australia; Taungurung; Cherokee Nation Citizen; Department of Biostatistics and Epidemiology, Hudson College of Public Health, University of Oklahoma Health Sciences Center, Oklahoma City, OK, USA; Elder from the Kungarakan tribal group and a member of the Iwaidja tribal group; Indigenous tobacco control advocate. Consultant to the Commonwealth Department of Health, Canberra, Australia

Publication and conference policies such as the Policy on the Tobacco Industry^1^ allow scientific societies and journals to accept publications and presentations that are funded in whole, or in part, by the Tobacco Industry and its affiliates. The argument is that, in these times of distrust and polarization of science, it is important to provide a forum for “good science” and “open scientific debate,” along with disclosures concerning funding, for the full range of views.^2–4^

However, Indigenous peoples are generally not comfortable with policies that give the Tobacco Industry, their affiliates and benefactors a seat at the table. Indigenous peoples continue to experience disproportionately high rates of commercial tobacco use and commercial tobacco-related death and disease.^5^ There is an inevitable conflict between the interests of Indigenous peoples and those of the Tobacco Industry and its affiliates^6^: for Indigenous peoples, our future lies in ridding ourselves of the physical, social, and spiritual harms caused by commercial tobacco use and nicotine addiction.

Many Indigenous peoples on Turtle Island and around the world have long had a sacred relationship with tobacco plants.^6^ Indigenous peoples have utilized ceremonial or sacred tobacco for gifting, prayer, and communicating with the Spirit World and the Creator.^7^ However, colonization—that is, the arrival of colonizers who settled and established control and dominance over the Indigenous peoples—altered and continue to affect Indigenous relations, cultures, ceremonies, sciences, and the sharing of knowledges.^6,7^ While some Indigenous peoples continue to use tobacco for ceremonial or sacred purposes,^7^ a significant change came with the commercialization of tobacco; selling back an appropriated and distorted culture to Indigenous peoples as consumers.^8^ Exploiting tobacco for profitability has led to a massive increase in commercial (ie, nonceremonial, nontraditional) industrialized tobacco use, with the inevitable and tragic impacts on health and wellbeing.

We argue that the notion that “good science” and “open scientific debate” should always be welcomed if it passes peer review is fundamentally flawed and that disclosure about funding sources does not provide sufficient protection from biases and commercial influences.^2–4^ The Society for Research on Nicotine and Tobacco (SRNT), Nicotine and Tobacco Research (NTR), and public health and tobacco control organizations generally should not partner with the Tobacco Industry, their affiliates and benefactors because, whether explicitly or implicitly, the Tobacco Industry supports and funds science that promotes its interests, specifically its commercial interests, over improving Indigenous health and wellbeing.^2,9^

## Engaging Membership to Eradicate an Industrialized Commercial Tobacco Epidemic

The Tobacco Industry has a long history of endangering Indigenous health and wellbeing, and that is continuing.^6^ Tobacco Industry approaches have intensified. There is an urgent need for a safer environment for Indigenous peoples that is free from Tobacco Industry influence that seeks to further their own interests.^6,10^ We are concerned not only with direct industry influence but via industry affiliates and benefactors, in particular industry-funded research.

As Indigenous people around the world are disproportionately targeted with commercial tobacco and nicotine marketing^11,12^ and are most significantly impacted by commercial tobacco-related harms,^5^ a fundamental aim of public health and commercial tobacco control must be to support Indigenous peoples to end exposure to and use of commercial tobacco and nicotine. This must be seen within the context of Indigenous models of health and wellbeing that connect physical, psychological, social, and spiritual health. This common commitment to protecting the health and wellbeing of our peoples is also consistent with the United Nations Declaration on the Rights of Indigenous Peoples (UNDRIP)^13^ and the World Health Organization’s Framework Convention on Tobacco Control (FCTC).^14^

**Figure 1.**
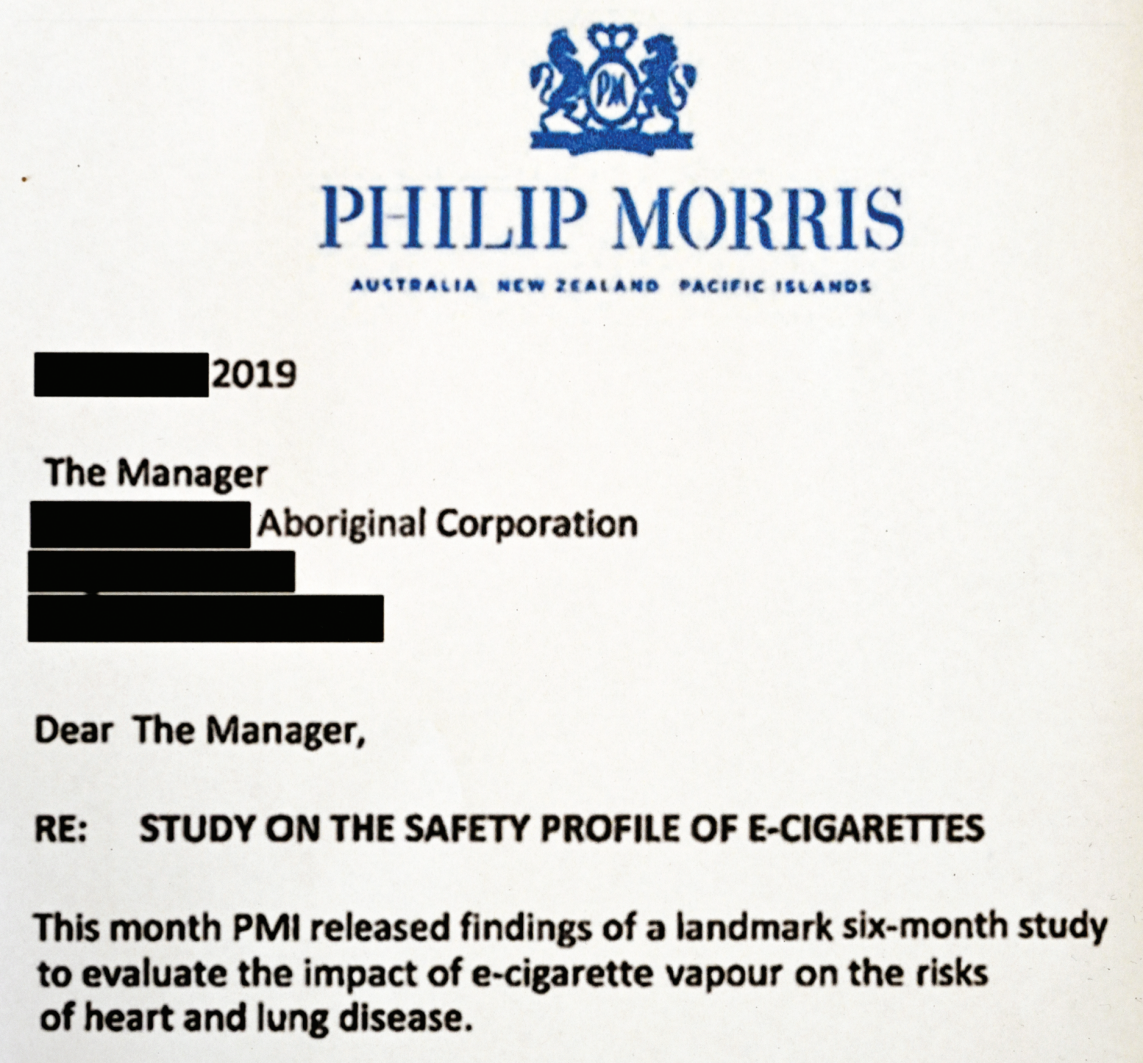
Letter from the Tobacco Industry to Indigenous organizations.

Indigenous peoples’ interests (and rights^13^) and our public health and commercial tobacco control initiatives to promote health are inherently at odds with the interests of the Tobacco Industry and their affiliates.^6^ The Tobacco Industry and its affiliates^9^ are required and incentivized to serve the “best interests” of the company. While it may also feel a need to reflect concerns about the triple bottom line (profit, people, and the planet),^15^ it takes a nuanced approach: profit appears to be prioritized, even if profit conflicts with people or planet.^15^ When claiming to consider the best interests of people and the planet—or other social goods—it has a long history of selecting the components that improve profits and widen the scope of the market (Figure 1).^15^

## Kei hopu tōu ringa ki te aka taepa, engari kia mau ki te aka matua (Māori proverb: Do not grasp the vine that hangs loose, but hold tight to the parent vine, anchored firmly below to Papa the earth and above to Rangi the sky)

There is indisputable evidence that the Tobacco Industry repeatedly and systematically interferes with legitimate scientific research.^2,9^ It uses research funding and industry-funded researchers, scientists, and consultants to suppress, delay, and impede the dissemination of knowledge, sowing confusion, and doubt, while generating opportunities to promote its own agenda.^2,9^ It has also used industry-funded findings to deceive consumers; undermined public health through manipulation, suppression, and misrepresentation of scientific data and poor research practices; and sought to undermine and distract attention from the science on smoking and smoking-related morbidity and mortality.^3,4^

Yet the industry continues to position itself as a strategic partner for public health, presenting itself as a scientific authority promoting new products as solutions to concerns about commercial tobacco and as a legitimate commentator on science and health research and policy.^6^ The industry’s characterization of itself as “socially responsible” belies the fact that the Tobacco Industry is itself the vector for the industrialized commercial tobacco epidemic and relies on a continued base of consumers of its products to maintain its existence. For example, the Tobacco Industry continues to invest in the combustible markets while in Australia alone, commercial tobacco is responsible for 37% of all Aboriginal and Torres Strait Islander deaths.^16^

## Coloniality and Unsafe Environments: “A Hawk That Lives in the City Is Still a Hawk” (Cree-Métis Saying)

Policies such as those outlined in Policy on the Tobacco Industry^1^ sideline Indigenous knowledges, sciences, research, and values and the health and wellbeing of our peoples, increasing profit for the Tobacco Industry.^6,17^ We argue that the focus on disclosures at an individual level may also neglect to protect against the possibility that the industry may seek to influence entire bodies of knowledge. For example, building an evidence base to support its agenda, as well as undermining and seeking to discredit legitimate research.

This is not our first rodeo. These approaches use common tools of colonization: oppress, divide, conquer based on racialized logics and, arguably and ultimately, use genocide, including cultural genocide and the adulteration and manipulation of sacred tobacco plants.^6,7,17^ This is why commercial tobacco has been described as a “modern form of colonization.” ^6^

The presence of the Tobacco Industry in the research environment creates an unsafe environment for Indigenous peoples and researchers. Indigenous peoples may feel silenced and unable to express their views, as they may be attacked by the industry.^18^ There is a fear of reprisals (eg, on social media and through legal threats and intimidation).^18^ We believe this perpetuates harms, especially for our emerging and community-based researchers.

It is not surprising that an increasing number of journal editors have made evidence-based decisions, in line with their principles and values, not to publish research funded either wholly or partly by the Tobacco Industry^19,20^ or research by authors who accept Tobacco Industry funding.^19,20^ Analysis of industry-funded research has repeatedly shown that the funding source shapes the study outcomes. This has been the case with a wide range of industries, including for example, with pharmaceuticals, medical devices, and food as well as commercial tobacco.^21^ Journal editors’ decisions recognize that peer review can play only a partial role in preventing industry manipulation of science, and the evidence base, since research misconduct and its impacts on study findings and conclusions are often impossible to detect. In our view, decisions not to publish research funded by the Tobacco Industry^19,20^ are consistent with UNDRIP^13^ and WHO FCTC Article 5.3^14^ and help to foster safer environments for Indigenous peoples.

## Conclusion and Recommendations

In dealing with Tobacco Industry-funded research, “scientific society” must follow the evidence and align the policy with UNDRIP and the WHO FCTC.^14^ We strongly recommend SRNT and NTR uphold the following principles:

1. *Ensure supportive and safe contexts*: Ongoing exploitation and manipulation of Indigenous peoples by the Tobacco Industry is an example of contemporary coloniality. For Indigenous researchers, allies, and colleagues, this creates unsafe environments and inhibits their ability to openly discuss and share their work. SRNT and NTR have a duty of care to ensure their safety is upheld, free from vested commercial interests. This is consistent with Article 5.3 of the FCTC.
2. *Support Indigenous leadership*: In (re)producing colonial practices of exploitation, the Tobacco Industry demonstrates its willingness to undermine Indigenous voices and communities. Indigenous leadership is not claimed by the individual, but endowed by wider Indigenous communities. The Society should actively support Indigenous leadership within the research community and the wider Indigenous community.
3. *Broaden the definition of “science,” evidence and knowledge*: Scientific evidence hierarchies tend to frame individualized studies (eg, RCTs, case–control) as the most “robust” forms of evidence. While they are important for individuals, the interventions associated with these studies often frame health within a euro-western medical perspective and offer little to address basic causes of smoking-related inequities such as structural/population level factors in society, and rooted in histories of racism, genocide, exploitation, and exclusion. The Tobacco Industry exploits this by attempting to focus attention on individualized biomedical studies, while scientifically ignoring, although commercially mining, structural inequities. Alternative research approaches, driven by Indigenous peoples as well as methodologies and methods are required to help broaden the definition of “science,” evidence and knowledges.
4. *Foster Indigenous research*: Principles 1–3 will encourage Indigenous-led research that frames the harms caused by commercial tobacco and understands how they can be addressed.

It is now the turn of the SRNT and NTR to make a choice that will ultimately define them. Will they be an asset to Indigenous health and wellbeing or another Tobacco Industry pawn?

## Supplementary Material

A Contributorship Form detailing each author’s specific involvement with this content, as well as any supplementary data, are available online at https://academic.oup.com/ntr.

ntab267_suppl_Supplementary_Data
